# Marine-Derived Angiogenesis Inhibitors for Cancer Therapy

**DOI:** 10.3390/md11030903

**Published:** 2013-03-15

**Authors:** Ying-Qing Wang, Ze-Hong Miao

**Affiliations:** Division of Antitumor Pharmacology, State Key Laboratory of Drug Research, Shanghai Institute of Materia Medica, Chinese Academy of Sciences, 555 Zu Chong Zhi Road, Zhangjiang Hi-Tech Park, Shanghai 201203, China; E-Mail: yqwang@jding.dhs.org

**Keywords:** marinenatural products, angiogenesis, protein kinase, cancer therapy

## Abstract

Angiogenesis inhibitors have been successfully used for cancer therapy in the clinic. Many marine-derived natural products and their analogues have been reported to show antiangiogenic activities. Compared with the drugs in the clinic, these agents display interesting characteristics, including diverse sources, unique chemical structures, special modes of action, and distinct activity and toxicity profiles. This review will first provide an overview of the current marine-derived angiogenesis inhibitors based on their primary targets and/or mechanisms of action. Then, the marine-derived antiangiogenic protein kinase inhibitors will be focused on. And finally, the clinical trials of the marine-derived antiangiogenic agents will be discussed, with special emphasis on their application potentials, problems and possible coping strategies in their future development as anticancer drugs.

## 1. Introduction

Angiogenesis not only plays an important role in physiological processes but is also involved in initiating and promoting several diseases such as cancer. As a hallmark of cancer [1], angiogenesis is considered as a powerful target to suppress tumor growth and metastasis. Several target proteins that are crucial for angiogenesis have been identiﬁed, *i.e.*, vascular endothelial growth factor (VEGFR)-related kinases [2], matrix metalloproteinases (MMPs) [3], methionine aminopeptidase (MetAP) [4], actin, microtubule [5], and histone deacetylases (HDACs) [6]. Using small molecules that inhibit angiogenesis-associated proteins has become a successful strategy for cancer therapy in the clinic [7].

Marine-derived natural products have been known for their huge diversity of chemical structures, and their unique structures are frequently associated with special mechanisms of action by which they may elicit unexpected biological activities. Therefore, increasing attention has been paid to the development of marine-derived anticancer drugs. Till now, two of four approved marine-derived drugs are anticancer drugs, *i.e.*, cytarabine [1969, approved by the Food and Drug Administration (FDA)] and ecteinascidin 743 (2007, approved by the European Agency for the Evaluation of Medicinal Products) [8,9]. As an important type of anticancer drugs, in recent years, marine-derived antiangiogenic agents have been widely investigated. At least 43 marine-derived natural products and their derivatives have been reported to display antiangiogenic activities, mediated by distinct or unknown molecular mechanisms. Among them, 10 entered either phase I, phase II or phase III clinical trials for cancer therapy. This review first provides an overview of these agents based on their potential distinct mechanisms/molecular targets, and then focuses on those that modulate kinase activities and those in clinical trials. 

## 2. An Overview of Marine-Derived Antiangiogenic Agents

**Figure 1:**
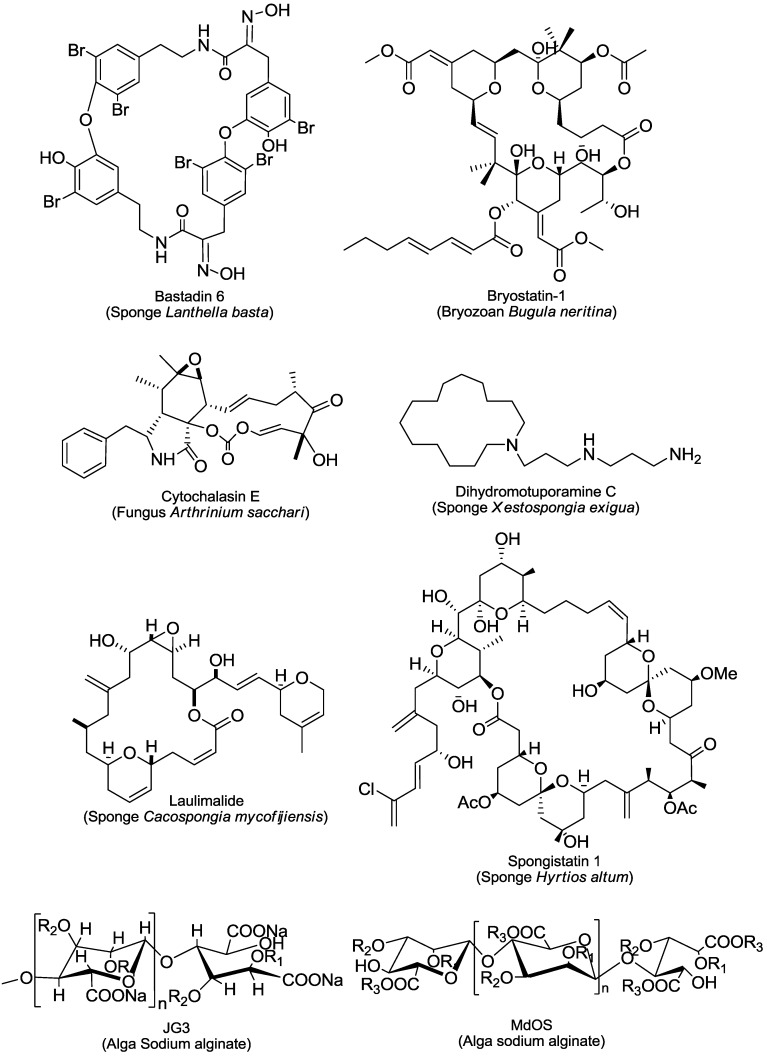
Structures and sources of macrocycles and saccharides. JG3,R_1_ = SO_3_Na, R_2_ = H or SO_3_Na, *n* = 3–9; MdOS, R_1_ = SO_3_Na, R_2_ = H or SO_3_Na, R_3_ = CH_2_CH(OH)CH_3_ or Na, *n* = 2–8.

**Figure 2:**
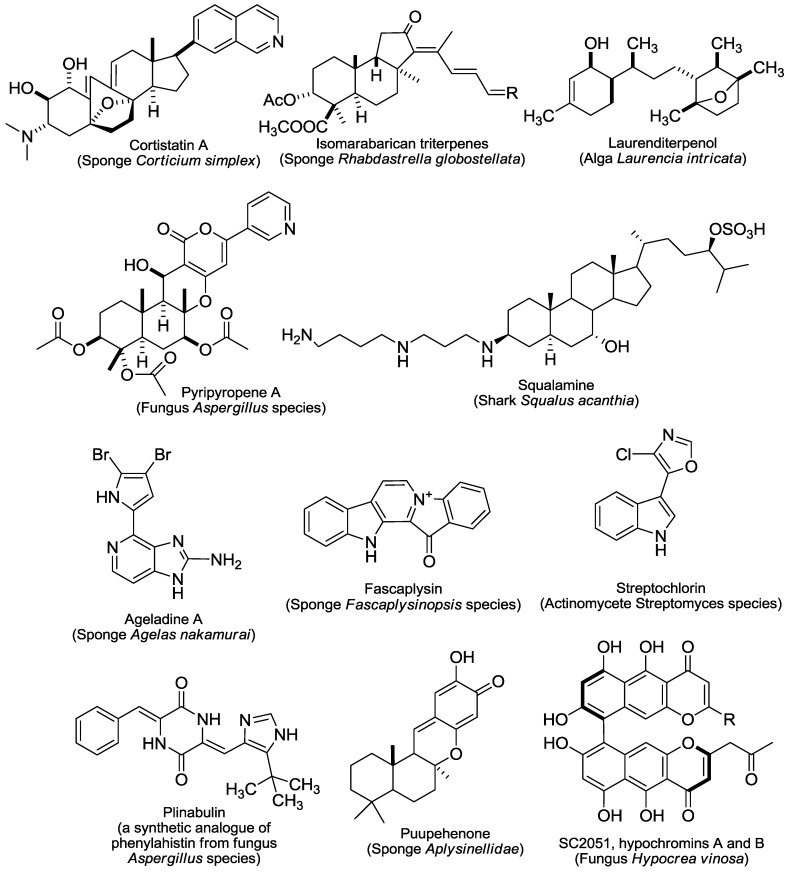
Structures and sources of terpenes, alkaloids and pyrones.SC2051, R = 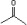;hypochromins A, R = 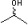; and hypochromins B, R = CH3.

**Figure 3:**
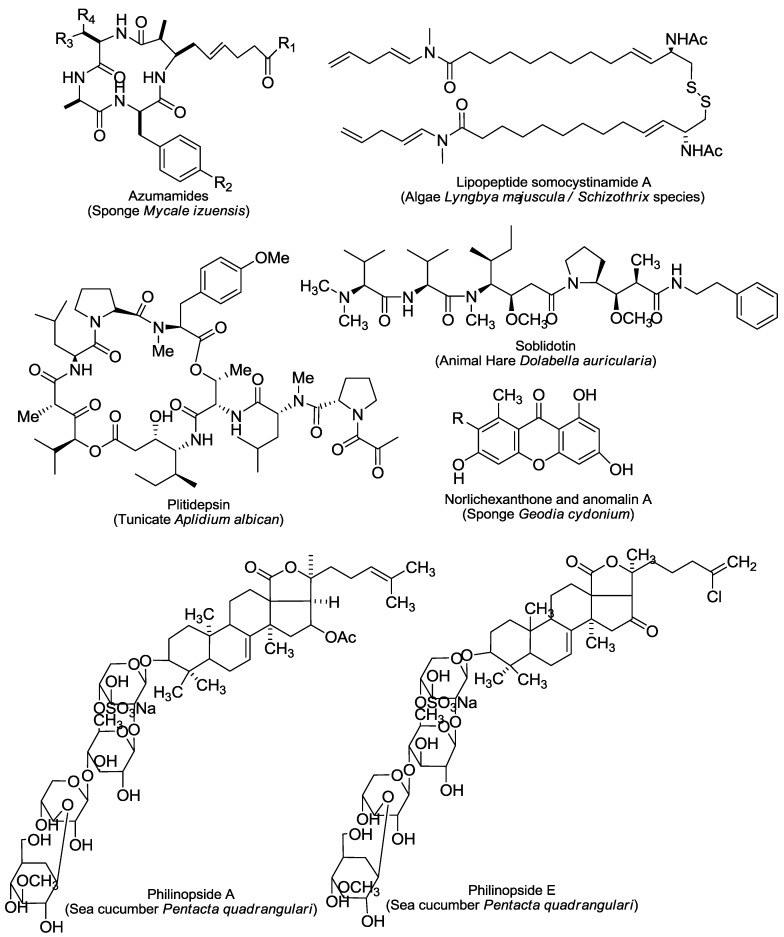
Structures and sources of peptides, saponins and xanthones. norlichexanthone, R = H; anomalin A, R = OH.

**Figure 4:**
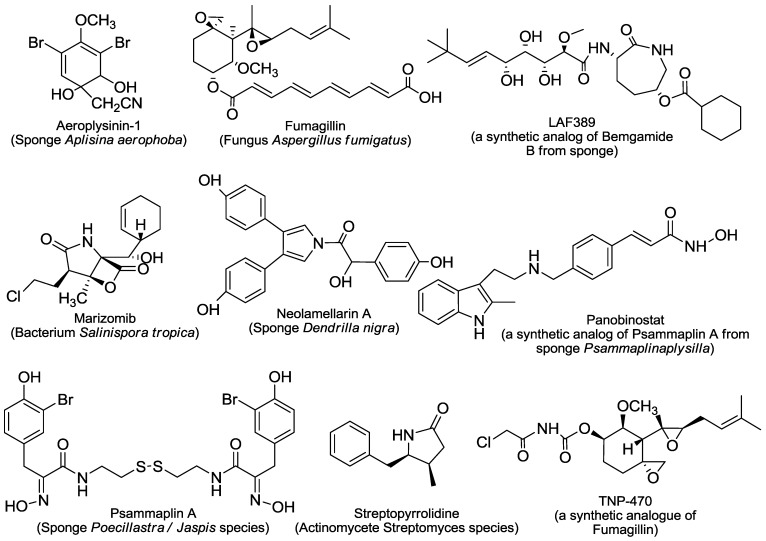
Structures and sources of other compounds.

The reported 43 marine-derived antiangiogenic agents have various sources (Figure 1, Figure 2, Figure 3, Figure 4). More than half of them (24) come from marine animals, including sponge, sea cucumber, bryozoan, tunicate, sea hare, and shark. Among them, 17 are from different sponges. The second important source is marine microorganisms including fungi, bacteria and actinomycetes that produce 11 agents. Marine phytoplanktons (mainly different algae) are their third largest source, from which 8 natural antiangiogenic agents come. This feature suggests that as a source of natural antiangiogenic agents, marine animals, especially sponges, have absorbed the most attention in the related investigation. It also shows that there are still tremendous opportunities to find new types of antiangiogenic agents from the ocean because the above-listed explored sources are just a small fraction of the marine resource that contains nearly 80% of all kinds of life on the Earth [9].

The great diversity of their chemical structures, partially due to their distinct sources, further characterizes those agents (Figure 1, Figure 2, Figure 3, Figure 4). Among them, there are six saccharides (JG3, MdOS, native fucoidan, oversulfation of fucoidon, SargA and sulfated galactan), six macrocycles (bastadin 6, bryostatin-1, cytochalasin E, dihydromotuporamine C, laulimalide and spongistatin 1), five terpenes (cortistatin A, isomarabarican triterpenes, laurenditerpenol, pyripyropene A and squalamine), four alkaloids (ageladine A, fascaplysin, plinabulin and streptochlorin), four pyrones (hypochromin A, hypochromin B, puupehenone and SC2051), four peptides (azumamides, plitidepsin, soblidotin and somocystinamide A), two saponins (philinopside A and philinopside E), two xanthones (anomalin A and norlichexanthone), nine of other structures (aeroplysinin-1, fumagillin, LAF389, marizomib, neolamellarin, panobinostat, psammaplin A, streptopyrrolidine and TNP-470), and one extract from shark cartilage (neovastat). The diverse chemical structures confer distinct activities and mechanisms of action to those agents. Here we roughly divide the reported 43 marine-derived antiangiogenic agents into five classes: Protein kinase modulators, cytoskeleton disturbing agents, HDAC inhibitors, MetAP inhibitors and others, based on their mechanisms/primary targets.

### 2.1. Protein Kinase Modulators

According to the difference in phosphorylating amino acids, protein kinases can be primarily classified into two types: tyrosine kinases (TKs) and serine/threonine kinases (S/TKs). TKs contain two large families: transmembrane receptor-linked kinases (RTKs) and cytoplasmic (non-receptor) tyrosine kinases (CTKs). VEGFR, platelet-derived growth factor receptors (PDGFR), fibroblast growth factor receptor (FGFR) and EGFR belong to RTKs while focal adhesion kinase (FAK), janus kinase (JAK) and sarcoma kinase (Src) are CTKs. Most of these kinases are frequently aberrantly activated in cancer and involved in tumor angiogenesis. The most famous S/TKs include protein kinase C (PKC) and cyclin-dependent kinases (CDKs). Of the 43 marine-derived antiangiogenic agents included in this present review, 14 are able to modulate protein kinases, which can be further divided into TK inhibitors (11) and S/TK modulators (3). These agents will be discussed separately in the second section.

### 2.2. Cytoskeleton Disturbing Agents

Microtubule and actin are the major structural compositions of cytoskeleton that is involved in many important cellular events including cellular shape, mitosis, movement, signaling transduction and substance transportation. Agents targeting microtubule such as taxol and vincristine are also clinically important anticancer drugs, and some of them show apparent antiangiogenic and/or antivascular properties [10]. Six marine-derived cytoskeleton disturbing agents (plinabulin, soblidotin, spongistatin 1, laulimalide, cytochalasin E and dihydromotuporamine C) have been shown to inhibit angiogenesis and/or to disrupt the established tumor vasculature. Among them, plinabulin (Figure 2) and soblidotin (Figure 3) entered phase II clinical trials.

Among the four microtubule inhibitors, three (plinabulin, soblidotin and spongistatin 1) inhibit tubulin polymerization while one (laulimalide) promotes it. These inhibitors have distinct binding sites on microtubule. Soblidotin (TZT-1027), a synthetic derivative of dolastatin-10 isolated from the Indian Ocean sea hare *Dolabella auricularia*, has two microtubule-binding sites of low and high affinity, partially overlapping the vinblastine-binding site of tubulin [11]. Plinabulin (NPI-2358), a synthetic analogue of phenylahistin (NPI-2350) isolated from marine fungus *Aspergillus* sp., interacts with the interfacial region of α- and β-tubulin, which partially overlaps with the colchicine binding site [12]. The binding site of spongistatin 1 (Figure 1) is unknown but different from that of vinblastine [13]. In contrast, the tubulin polymerization agent laulimalide (Figure 1) binds to the exterior of the microtubule on β-tubulin, a binding site different from the taxol-binding site [14,15]. Despite these differences, all the 4 inhibitors possess potent cytotoxicity in various tumor cells *in vitro* and *in vivo*. They also cause cell cycle arrest in the G_2_/M phase and induce apoptosis [16,17,18,19]. And also due to the differences in their binding to tubulin or their unique structural characteristics, some of them such as plinabulin and laulimalide are highly effective even in cancer cells with resistance to taxol or multidrug resistance (MDR) [14,20].

These four microtubule inhibitors show potent angiogenesis inhibitory activity in a variety of angiogenesis experimental models (Table 1). Some inhibitors could prominently inhibit angiogenesis at much lower concentrations/doses than cytotoxic ones [21,22], suggesting the involvement of additional mechanism(s) apart from their microtubule inhibition. Actually, spongistatin 1 could inhibit the enzymatic activity and intracellular translocation of PKCα that is an essential kinase in angiogenesis [21]. Laulimalide could block integrin-associated signaling pathways through suppressing the phosphorylation of FAK and paxillin and the association of VEGFR-2 with FAK and heat shock protein 90 (Hsp90), although it did not inhibit the phosphorylation of VEGFR-2 [22]. In addition to its antiangiogenic activity, soblidotin shows its antivascular effect at a little higher concentrations, which might be independent of its microtubule inhibition [23,24]. Of note, plinabulin demonstrates significant vascular disrupting activity by acting on established tumor blood vessels. This activity has been considered to result from its inhibiting tubulin polymerization, which impairs cytoskeletal function and leads to selective tumor vascular endothelial architectural destabilization and vascular collapse [25]. 

**Table 1:** Assays used to test for angiogenesis inhibitory activity of agents reviewed in this paper. One or more models were used in the cited studies for the discussed agents.

***In vitro* assays:**
▪ Proliferation, migration, adhesion, monolayer permeability, or tube formation of endothelial cells [human umbilical vein endothelial cells (HUVECs), human microvascular endothelial cells (HMECs), human umbilical artery endothelial cells (HUAECs), or bovine aortic endothelial cells (BAE)]
***Ex vivo* assays:**
▪ Sprout outgrowth in rat or chicken aortic ring cultures
***In vivo* assays:**
▪ Chicken chorioallantoic membrane (CAM) assays ▪ Choroidal neovascularization membranes (CNVMs) assays in rats ▪ Corneal angiogenesis assays in mice or rabbits ▪ Matrigel plug assays in mice ▪ Tumor vessel density assays by immunohistochemistry in xenografts ▪ Systemic angiogenesis assays in zebrafish

Both cytochalasin E (Figure 1) and dihydromotuporamine C (Figure 1) target actin. However, cytochalasin E leads to actin depolymerization only at relatively high concentrations. Cytochalasin E at lower concentrations preferentially inhibits endothelial cell proliferation without disrupting actin stress fibers [26]. It also suppresses the phosphorylation of FAK, which may be critical to its antitumor activity [27]. Interestingly, cytochalasin E was reported to activate PKC, leading to the induction of interleukin-8 production and the up-regulation of CD54 [28,29]. Dihydromotuporamine C is one of nine members in the motuporamines family (motuporamine A–I). It could increase the thickness and number of cytoplasmic actin stress fibers, induce the formation of new stress fibers and large focal adhesion complexes, activates the small GTP-binding protein Rho, and stimulate the Rho-dependent sodium-proton exchanger activity. Its ability to activate Rho has been considered to be one important determinant of its anti-invasive activity [30]. As other members in this family, dihydromotuporamine C shows very low cytotoxicity or cell proliferation inhibition. Both cytochalasin E and dihydromotuporamine C displayed antiangiogenesis activity in different *in vitro* and *in vivo* models (Table 1) [26,31].

### 2.3. HDAC Inhibitors

HDACs are the enzymes that remove acetyl groups from target proteins that regulate their activity. Eighteen human HDACs have been found that can be divided into four classes (class I–IV). HDACs are important anticancer targets, and 2 HDAC inhibitors were approved by FDA (vorinostat in 2006 and romidepsin in 2009) for treating cutaneous T-cell lymphoma [32]. Three marine-derived HDAC inhibitors including panobinostat (Figure 4), azumamides A–E (Figure 3) and psammaplin A (Figure 4) have been reported to inhibit angiogenesis. Panobinostat (LBH589), one of the most potent HDAC inhibitors, can inhibit all known HDACs, which leads to G_2_/M cell cycle arrest and proliferation inhibition [33]. At noncytotoxic concentrations, panobinostat also inhibits the phosphorylation of protein kinase B (AKT) and extracellular signal-regulated kinase (ERK) 1/2, and the expression of the chemokine receptor CXCR4. Panobinostat exerts antiangiogenesis activity evidenced by its inhibition of endothelial tube formation and Matrigel invasion, and its reduction of angiogenesis in PC-3 xenografts in mice [34]. Azumamides A–E are HDAC inhibitory cyclic tetrapeptides isolated from the marine sponge *Mycale izuensis*. Azumamide A displayed significant inhibition of vascular formation [35]. The data from an *in vitro* vascular organization model using mouse induced pluripotent stem (iPS) cells revealed that the antiangiogenic activity of azumamides was closely correlated with their HDAC inhibition [36]. Psammaplin A from marine sponges potently inhibits HDAC enzyme (an IC_50_ value of 4.2 nM) [37] and relatively weakly suppresses mammalian aminopeptidase N (an IC_50_ value of 18 μM) [38]. Psammaplin A causes upregulation of the CDK inhibitor p21^WAF1^ and downregulation of pRb, cyclins and CDKs [39]. Psammaplin A has potent antiproliferative activity against various tumor cells and significant antiangiogenesis effects in different vascular formation models (Table 1) [38,40].

### 2.4. MetAPs Inhibitor

MetAPs are the essential metalloproteins that remove the initiator NH_2_-terminal methionine from nascent proteins. Two types of MetAP enzymes, MetAP-1 and MetAP-2, have been reported. MetAP-2 has been investigated as anticancer and antiangiogenesis targets since the discovery that the marine natural product fumagillin (Figure 4) irreversibly inhibits MetAP-2 (rather than MetAP-1), impairs angiogenesis, and elicits anticancer activity [41,42,43]. Fumagillin covalently binds to His231 of MetAP-2 that is located in the active site of the enzyme and thus prevents the binding of substrates to MetAP-2 [43]. Nevertheless, how MetAP-2 regulates angiogenesis and how fumagillin inhibits it remain to be clarified. TNP-470 (Figure 4) is a semisynthetic analogue of fumagillin with 50-fold increase in inhibiting neovascularization and angiogenesis. TNP-470 potently and selectively acts on endothelial cells. It inhibits endothelial proliferation *in vitro* at a concentration 3 logs lower than that required to inhibit the proliferation of ﬁbroblasts and tumor cells [44]. TNP-470 was also reported to inhibit FGF1-stimulated angiogenesis both *in vitro* and *in vivo* through the binding to the cytoplasmic domain of the FGF1 receptor [45]. 

Different from fumagillin, the marine natural products bengamides inhibit both MetAP-1 and MetAP-2 with similar potency. Different bengamides can bind to the active center of the enzymes but in differential binding modes [46]. LAF389 (Figure 4) is a synthetic analogue of bengamide B. LAF389 significantly inhibits tumor growth and proliferation, apparently by means of MetAP inhibition [47,48]. LAF389 demonstrates a marked inhibition of tumor vascularization in animal experiments. However, no antiangiogenic activity data was obtained from patients owing to the early discontinuation of its phase I clinical trials [49]. 

### 2.5. Others

There are additional marine natural products that show antiangiogenic activity in different models (the tube formation assay was used most widely) (Table 1) but via unique or unknown mechanisms. Some of them were reported only in few studies. For example, puupehenone (Figure 2) was revealed to inhibit the tube formation of endothelial cells and the *in vivo* angiogenesis in the CAM assay [50]; streptochlorin (Figure 2) suppresses the invasion and VEGF-stimulated tube formation of endothelial cells [51]; the lipopeptide somocystinamide A (Figure 3) impairs the proliferation and tube formation of endothelial cells and inhibits the angiogenesis in zebrafish or CAM models [52]; bastadin 6 (Figure 1), pyripyropenes A (Figure 2) and isomarabarican (Figure 2) highly selectively inhibit the proliferation, migration and tube formation of endothelial cells, and bastadin 6 also blocks the VEGF- or bFGF-induced *in vivo* angiogenesis of mice corneas [53,54,55]; and streptopyrrolidine (Figure 4) significantly blocks the tube formation of endothelial cells at a non-cytotoxic concentration [56]. Several saccharides mainly from marine algae such as SargA from *Sargassum stenophyllum* [57], sulfated galactan from *Codium cylindricum* [58] and a series of fucoidans from different brown algae species, also show antiangiogenic activity to different degrees [59,60]. In addition, there are still some other marine-derived agents that can interfere with angiogenesis-related signaling although no direct antiangiogenesis activity has been reported yet. Two examples are laurenditerpenol (Figure 2) and neolamellarin A (Figure 4), both of which can inhibit the activation of hypoxia-inducible factor (HIF)-1, a critical event in tumor neoangiogenesis [61,62].

In contrast, another three antiangiogenesis agents have been investigated in more detail and all of them entered clinical trials. The marine-derived depsipeptide plitidepsin (dehydrodidemnin B, aplidine) (Figure 3), the aminosterol squalamine (Figure 2) and the proteasome inhibitor marizomib (NPI-0052, salinosporamide A) (Figure 4) all show the antiangiogenic effect in multiple *in vitro* and *in vivo* angiogenesis models (Table 1). Plitidepsin could block the secretion of VEGF by tumor cells and the production of MMP by endothelial cells [63], and reduce the expression of anigiogenesis-related genes including VEGF and HIF-1 [64]. Its antiangiogenic activity contributed to its antitumor effects [65]. However, the primary target(s) of plitidepsin remain to be discovered. Squalamine alters intracellular pH and impedes the growth factor signaling, possibly by selectively inhibiting the sodium-hydrogen antiporter exchanger of the endothelial cell surface. It exerts the greatest effect on newly emerging vessels with no appreciable effect on unstimulated endothelial cells [66,67,68]. Additionally, squalamine shows potential antitubercular and antimicrobial activities [69,70]. Marizomib inhibits 20S proteasome by covalently modifying its active site threonine residues and exhibits therapeutic activity against a wide variety of tumors [71]. Marizomib significantly interferes with several angiogenesis-associated factors, including VEGF-dependent migration [72], TNF-α-induced VEGF expression [73], and downregulation of HIF-1α and HIF-2α proteins and VEGF secretion [74], inhibition of both constitutive and inducible prosurvival transcription factor NF-κB [73,74].

## 3. Marine-Derived Protein Kinase Modulators with Antiangiogenic Activity

Two types of the marine-derived protein kinase modulators, *i.e.*, S/TK modulators and TK inhibitors have been reported to have antiangiogenic activity

### 3.1. S/TK Modulators

S/TKs are a large protein kinase family, including PKC, CDKs and Rho-associated, coiled-coil containing protein kinase (ROCK). Several S/TK-targeted marine-derived natural products showed antiangiogenic activity in different models (Table 1). Of them, bryostatin-1 has entered phase II clinical trials. 

#### 3.1.1. The PKC Activator Bryostatin-1

The PKC family contains at least 12 isozymes characteristic of different structures, functions, subcellular localization, and substrate specificity. Most PKC activators such as the phorbol ester 12-*O*-tetradecanoylphorbol-13-acetate (TPA) are potent tumor promoters. In contrast, the PKC activator bryostatin-1 (Figure 1), a monocyclic lactone originally derived from a marine bryozoan and now available by synthesis, shows unique anticancer activities with little tumor promotion [75]. Similar to TPA, bryostatin-1 can activate almost all PKC isozymes [76], indicating that its anticancer effects cannot be elicited only through its PKC activation. Actually, there is an important difference between bryostatin-1 and TPA that is their distinct impacts on the proposed tumor suppressor PKCδ. TPA downregulates it whereas bryostatin-1 does not, even at high concentrations [77,78]. Such a difference might result from their distinct binding modes: TPA selectively binds to the C1B domain but bryostatin-1 binds to both C1A and C1B domains of PKCδ [75]. Nevertheless, the exact molecular mechanisms that mediate bryostatin-1’s unique anticancer activities still need further investigating. Bryostatin-1 can potentially inhibit tumor invasion, angiogenesis, cell adhesion, and limit the development of MDR by regulating PKC activity [79,80].

#### 3.1.2. The CDK4 Inhibitor Fascaplysin

The marine natural product fascaplysin (Figure 2) inhibits CDK4 with more than 1400-fold selectivity over its close homolog CDK2. The positive charge in its structure is crucial for this high selectivity [81]. Fascaplysin can block VEGF, inhibit proliferation and induce apoptosis of human umbilical vein endothelial cells (HUVECs) [82]. These activities could contribute to its antiangiogenic effects shown in multiple *in vitro* and *in vivo* experimental models (Table 1) [83,84]. 

#### 3.1.3. Cortistatin A

Cortistatins (Figure 2) are a family of eleven steroidal alkaloids isolated from the marine sponge *Corticium simplex*. Of them, cortistatin A shows the most potent antiangiogenic activity. It could selectively inhibit the proliferation, migration and tube formation of HUVECs [85], which might be associated with its suppression of the phosphorylation of the unidentiﬁed 110 kDa protein in HUVECs [86] and some kinases such as ROCK, CDK8 and CDK11 [87]. The dimethylamino group and isoquinoline unit in the side chain of cortistatin A could be crucial for its antiangiogenic and kinase inhibitory activities [86]. 

### 3.2. TK Inhibitors

Numerous TKs such as VEGFR, EGFR, PDGFR and FGFR play critical roles in tumor angiogenesis, which are the major target (s) of most small-molecule angiogenesis inhibitors in the clinic including sorafenib and sunitinib [7]. As yet, however, only 11 TK-targeted marine-derived angiogenesis inhibitors have been reported, not so many as expected. Among them, neovastat (AE-941) entered phase III clinical trials but failed unfortunately, and the others are still in very early investigation stages.

#### 3.2.1. Neovastat

The antiangiogenic activity of neovastat, an extract from shark cartilage, has been widely investigated. Neovastat inhibits CAM vascularization and Matrigel-induced angiogenesis [88]; it competitively suppresses the VEGF-dependent phosphorylation of VEGFR, the proliferation of endothelial cells, and VEGF-induced vascular permeability in mice [89]. Neovastat could also inhibit MMP-2 [90] but stimulate tissue plasminogen activator (tPA) [91] and activate caspase-mediated apoptotic pathways in endothelial cells [92]. In addition, neovastat has significant antitumor and antimetastatic activity [93]. Although its phase II clinical trials were reported to produce positive therapeutic effects, subsequent 2 phase III clinical trials of neovastat failed in either stage III non-small cell lung cancer or metastatic renal cell carcinoma [94,95,96]. Moreover, “Sharks don’t get cancer” has been found to be not the case [97]. Therefore, the development of neovastat as an anticancer drug is likely to be ended.

#### 3.2.2. Aeroplysinin-1

Aeroplysinin-1 (Figure 4) is a marine-derived brominated tyrosine metabolite that has antibacterial, antiparasitic, anticancer and antiangiogenic activities. Using different *in vitro* and *in vivo* models (Table 1), aeroplysinin-1 has been demonstrated to inhibit the key steps of angiogenesis [98]. Its antiangiogenic activity would be associated with its selective apoptotic induction in endothelial cells via the intrinsic apoptosis pathway [98]. This selectivity may result from its significant inhibition of EGFR [99] and VEGFR-2 [100]. Additionally, new synthetic derivatives of aeroplysinin-1 demonstrate promising antiangiogenic potentials [100]. 

#### 3.2.3. Philinopside A and Philinopside E

Both are sulfated saponins isolated from the sea cucumber *Pentacta quadrangulari*. Philinopside A and E (Figure 3) displayed their antiangiogenic activity including inhibition of the proliferation, migration and tube formation of endothelial cells and the vascularization in the rat aorta ring culture assay, the CAM assay or mouse tumor models. The angiogenesis inhibition could result from their suppression of angiogenesis-related RTKs including VEGFR, FGFR-1, PDGFR, EGFR and their related signaling transduction [7,101,102].

#### 3.2.4. Ageladine A

The brominated pyrrole-imidazole alkaloid Ageladine A (Figure 2) was first isolated from the marine sponge *Agelas nakamurai* but now can be obtained by synthesis using different methods [103,104,105,106,107]. Ageladine A exhibited *in vitro* and *in vivo* antiangiogenic activity (Table 1), which was initially considered to be associated with its MMP inhibition [108] but subsequently confirmed to result from its selective inhibition of kinases including dual specificity tyrosine-phosphorylation-regulated kinase (DYRK) 1A, DYRK2, tyrosine kinase 2 (TYK2) and yeast Sps1/Ste20-related kinase 4 (YSK4) [103]. One of the most impressive features of ageladine A and some of its synthetic analogues is their highly selective angiogenesis inhibition because no cytotoxicity was found in the National Cancer Institute (NCI) panel of 60 human cancer cell lines at their antiangiogenic concentrations. In addition, ageladine A is also a pH-sensitive fluorescent dye that may be used for imaging [109,110].

#### 3.2.5. Saccharides

Several marine-derived saccharides, mainly from sea algae, have been reported to inhibit tumor angiogenesis, which is related to their TK inhibition. The semi-synthesized oligomannurarate sulfate JG3 (Figure 1) shows significantly antiangiogenic and antimetastatic activities. On one hand, JG3 impairs the activity of heparanase via binding to its KKDC (KKFKNSTYSRSSVDC amino acids) and QPLK (QPRRKTAKMLK amino acids) domains; on the other, JG3 reduces the bFGF-induced phosphorylation of bFGFR and ERK1/2 [111]. In contrast, another semi-synthesized oligosaccharideMdOS (Figure 1) (Marine-derived Oligosaccharide Sulfate) exerts its antiangiogenic effect that is dependent on its broad-spectrum TK inhibition on human epidermal growth factor receptor-2 (HER2), EGFR, VEGFR, PDGFR, c-Kit, FGFR1 and c-Src. MdOS acts as an ATP-competitive inhibitor of these TK enzymes [112]. 

Sulfated polysaccharides such as fucoidans and SargA were shown to modulate angiogenesis. Different fucoidans showed totally different effects on angiogenesis, which varied according to their molecular weight and sulfated degree. Low molecular weight fucoidans (4–9 kDa) were found to stimulate angiogenesis in different assays [113,114,115]; middle molecular weight fucoidans (15–20 kDa) could enhance HUVEC migration but did not inhibit HUVEC tube formation; and natural fucoidan had high molecular weight (30 kDa) and antiangiogenic properties shown by inhibition of the proliferation, migration and tube formation of endothelial cells and vascular network formation [59,116,117]. On the other hand, oversulfated fucoidan inhibited angiogenesis (the bFGF-induced migration and tube formation of HUVECs) apparently more potently than natural or desulfated fucoidan [118,119]. SargA, a sulfated heteropolysaccharide extracted from the brown marine alga *Sargassum stenophyllum*, was also reported to exhibit antiangiogenic activity *in vitro* and *in vivo* (Table 1) [57,120]. The antiangiogenic mechanisms of these sulfated polysaccharides are likely associated with their interfering with the phosphorylation of bFGFR, ERK1/2 or FAK [57,120,121].

#### 3.2.6. Anomalin A and Norlichexanthone

The xanthones anomalin A and norlichexanthone (Figure 3) can be isolated from the sponge-derived fungus *Arthrinium* sp. Both were shown to inhibit the proliferation of cancer cells and the activity of multiple protein kinases such as anaplastic lymphoma kinase (ALK), AMPK-related protein kinase (ARK5), Aurora-B, insulin-like growth factor-1 receptor (IGF1-R), pim kinase-1 (PIM1), polo-like kinase 1 (PLK1), PKC-related kinase 1 (PRK1), Src and VEGFR-2, but anomalin A was more potent and had a different spectrum of action [122,123]. This may be one of the reasons why anomalin A could inhibit VEGF-A dependent endothelial cell sprouting whereas norlichexanthone could not [123].

#### 3.2.7. SC2051 and hypochromins A and B

SC2051 and hypochromins A and B (Figure 2), all with bis (naphtho-γ-pyrone) skeletons, were isolated from the marine-derived fungi *Hypocrea Vinosa*. All three compounds showed tyrosine kinase inhibitory activity and could elicit antiangiogenic effects in assays for the proliferation, migration, and tube formation of endothelial cells [124].

## 4. Clinical Trials of Marine-Derived Angiogenesis Inhibitors for Cancer Therapy

Ten marine-derived angiogenesis inhibitors entered clinical trials for cancer therapy, but 5 of them failed in different stages. The phase III clinical trials of the shark cartilage extract neovastat [94,95,96] and the phase II clinical trials of the tubulin inhibitor soblidotin [125,126] failed due to the lack of the expected therapeutic efficacy. The development of MetAPs inhibitors LAF389 and TNP-470 was also stopped in either phase I or phase II due to the severe toxicities or the lack of positive therapeutic effects [49,127,128]. In addition, although the aminosterol squalamine showed tolerable toxicity in phase I/IIA clinical trials [129], there have been no reports about its clinic development in recent ten years. Therefore, we here only discuss the clinical data of the other five marine-derived angiogenesis inhibitors, *i.e.*, bryostatin-1, panobinostat, plitidepsin, marizomib and plinabulin. 

### 4.1. The PKC Activator Bryostatin-1

Since 1993, over 80 separate phase I and II clinical trials for cancer therapy using bryostatin-1 alone or in combination with conventional anticancer drugs have been conducted [8]. Different types of human cancers have been investigated in these clinical studies, including chronic lymphocytic leukemia, non-Hodgkin lymphoma, multiple myeloma, melanoma, renal cell carcinoma, colorectal cancer, soft tissue sarcoma, head and neck cancer, ovarian cancer, cervix carcinoma, gastric cancer, gastroesophageal cancer, esophageal cancer and pancreatic carcinoma [79,130,131,132,133,134,135,136,137,138,139,140,141,142,143,144,145,146,147,148,149,150]. From the results of its clinical trials, several important conclusions can be drawn: (**1**) Myalgia is its main human dose-limiting toxicity with the occurrence of 10%–87.5% found in phase II trials [151]. Although the precise mechanism of bryostatin-1-triggered myalgia remains unknown, it could be manageable by some measures such as exercise, taking analgesics, and dose control. Nevertheless, myalgia is still an important reason for ending clinical studies [151]. (**2**) Bryostatin-1 alone failed to produce adequate therapeutic effects in almost all examined hematological malignancies and solid tumors. (**3**) However, its combination with various conventional anticancer drugs seems encouraging. In particular, the combination of bryostatin-1 with paclitaxel, cisplatin or vincristine in phase II clinical studies produced promising therapeutic effects [146,147,148,150]. (**4**) The important characteristics of bryostatin-1 in these combinations might include its circumvention of tumor MDR and no or limited overlapping toxicities with the conventional anticancer drugs. Therefore, bryostatin-1 is possible to prevent or delay the occurrence of tumor MDR to the combined drug (s) such as paclitaxel or vincristine [148]. On the other hand, the toxicities of the combinations would not increase compared with those of their separate uses [75]. (**5**) As a PKC activator, bryostatin-1 is possible to act as a tumor promoter to accelerate the growth of some tumors although it has little tumor-promoting activity [75,152]. To reduce this risk, it may need to develop appropriate molecular biomarkers for the selection of patients and the therapeutic surveillance in its clinical studies and potential uses. 

In addition, although the antiangiogenic activity of bryostatin-1 was shown in preclinical studies, it was not reported in any clinical trials. So it could not be concluded whether the antiangiogenic activity practically contributes to the clinical anticancer effects of bryostatin-1. According to the present clinical results, the clinical development of bryostatin-1 is faced with several major challenges, including how to reduce its toxicity (mainly myalgia), increase its therapeutic efficacy (mainly through combination with other anticancer drugs), and develop proper molecular biomarkers for the selection of patients and the therapeutic surveillance. Another problem is its very limited yield from natural resources. Moreover, it is also hard to be synthesized [75]. However, because of its unique PKC modulation and anticancer activity, more potent analogues of bryostatin-1 deserve testing in the clinical arena. 

### 4.2. The HDAC Inhibitor Panobinostat

The marine-derived angiogenesis inhibitor panobinostat is among the most potent HDAC inhibitors [153]. It is undergoing extensive phase I and phase II clinical trials with 114 clinical studies [154]. Panobinostat is orally or intravenously available [153]. The major toxicities of panobinostat in humans are hematologic (including thrombocytopenia, neutropenia, anemia and leukopenia), fatigue, and gastrointestinal (nausea and diarrhea) but generally manageable [155,156,157,158,159,160,161,162]. Although phase II clinical trials of panobinostat have been conducted in solid (e.g., renal and pancreatic) cancers, the reported positive responses to its single-agent therapy have been predominantly observed in advanced hematologic malignancies including T-cell lymphoma, Hodgkin lymphoma, Waldenström macroglobulinemia, and myelodysplastic syndrome [155,156,157,158,159,160,161,162], showing a similar clinical anticancer spectrum to other HDAC inhibitors. However, the response of multiple myeloma to panobinostat monotherapy was just modest [156,157]. The combination of panobinostat with other anticancer drugs could expand its potential clinical applications. Its combination with paclitaxel and carboplatin reported in a phase I study [163] was well tolerated. In contrast, the combination of panobinostat, bevacizumab and everolimus was not tolerable [164]. The results are interesting because all panobinostat, paclitaxel and carboplatin can inhibit bone marrow but both bevacizumab and everolimus have very limited hematologic adverse effects. The results suggest that the toxicity of these combinations may have additional determinants and careful selection of its combined drugs is very important. The results also suggest that in addition to histone hyperacetylation, other biomarkers more relevant to the clinical therapeutic efficacy and toxicity of panobinostat are urgently needed [165].

### 4.3. The Cyclic Depsipeptide Plitidepsin

Plitidepsin is undergoing phase I and phase II clinical studies and its pivotal randomized phase III trial is also ongoing in patients with relapsed/refractory multiple myeloma [166,167]. Phase I results show that plitidepsin has a unique toxicity profile that is similar in children and adult cancer patients [168]. Its main dose-limiting toxicities are muscular and hepatic toxicities. The most common muscular toxicities are myalgia and asthenia with unknown mechanisms, and co-treatments with l-carnitine can prevent them or accelerate their recovery [168,169,170]. Its hepatic toxicities are transaminase increases but generally reversible. Other toxicities of plitidepsin include fatigue, pyrexia, skin rash, abdominal pain, nausea, vomiting and diarrhea. Of note, plitidepsin does not significantly inhibit bone marrow at pharmacological concentrations [168,170]. Moreover, it does not seem to produce cross-resistance between plitidepsin and other conventional anticancer drugs [171]. These two characteristics are important for they provide a critical basis for its clinical combination designs with the drugs that are myelosuppressive and/or sensitive to drug resistance mechanisms. 

Phase II studies with plitidepsin monotherapy further confirmed its favorable safety profile, but did not demonstrate significant clinical anticancer activity in solid tumors such as unresectable advanced medullary thyroid carcinoma [172], refractory advanced malignant melanoma [173], pretreated small cell lung cancer [174] and pretreated non-small cell lung cancer [175]. In contrast, the recently released results of a phase II study with single-agent plitidepsin revealed its clinical activity with an overall response rate of 20.7% (6 of 29; 2 complete responses and 4 partial responses) in patients with non-cutaneous peripheral T-cell lymphoma [176]. However, no responses were observed to other non-Hodgkin’s lymphoma including B-cell lymphoma in the same study. Plitidepsin showed synergistic activity with rituximab in preclinical models of diffuse large cell and Burkitt lymphoma [166]. Its combination with dexamethasone in a phase II clinical trial also displayed therapeutic efficacy though limited [177]. Therefore, future clinical development of plitidepsin might predominantly test for its therapeutic efficacy against hematological malignancies, preferentially through combination with other anticancer drugs.

### 4.4. The Proteasome Inhibitor Marizomib

Preclinical data indicate that marizomib inhibits all three enzymatic activities of the proteasome with preferential inhibition of the chymotrypsin-like and trypsin-like proteasome enzymes. Therefore, marizomib could overcome the resistance to bortezomib that selectively inhibits the chymotrypsin-like activity of the proteasome. In addition, this broader spectrum of enzymatic activity inhibition is also likely to confer a differential anticancer spectrum and a different toxicity profile to marizomib. This unique feature provided the basis for the clinical development of marizomib although bortezomib has been approved for clinical uses [178]. Marizomib is undergoing phase I clinical trials. The results available indicate its safety and preliminary anticancer activity. The most common adverse events of marizomib observed in these clinical studies were fatigue, nausea, diarrhea, vomiting, and dysgeusia/anorexia. Its dose-limiting toxicity could be transient hallucinations (visual imprints when eyes closed) and dizziness/unsteady gait. Notably, marizomib did not cause myelosuppression (neutropenia and thrombocytopenia) or peripheral neuropathy that could be related to bortezomib. In addition, the combination with the HDAC inhibitor vorinostat did not enhance the toxicity of marizomib but could synergistically increase its therapeutic efficacy in patients with melanoma, pancreatic and lung cancer [179,180,181]. With marizomib monotherapy, stable disease was observed in patients with multiple myeloma, melanoma, cervical, colorectal, hepatocellular, adenoid cystic, granulosis cell or ovarian carcinoma [180,182]. Nevertheless, marizomib has a very short half-life of 4–10 min with clearance at 3–15 L/min in patients, indicating that it is necessary to carefully adjust the administration schedule of marizomib so as to provide enough drug exposure to achieve clinical benefit [180,183]. Taken together, marizomib is well tolerated with a unique toxicity profile and has demonstrated preliminary therapeutic effects, which warrants its further clinical development. 

### 4.5. The Tubulin Inhibitor Plinabulin

The tubulin inhibitor plinabulin is a vascular disrupting agent, two phase I trials of which have been reported. The first one was conducted using single-agent plinabulin in 38 patients with advanced solid tumors (including colorectal, pancreatic, breast, renal thyroid, adrenocortical or prostate carcinoma) or lymphomas (diffuse large cell non-Hodgkin’s lymphoma) [184]. In contrast, the second one tested the combination of plinabulin with docetaxel in 13 patients with advanced non-small cell lung cancer, gastrointestinal stromal tumor, liposarcoma or melanoma [25]. The results showed its favorable safety profile with fatigue, tumor pain, nausea, diarrhea and vomiting as the most common but manageable adverse events. Fever, tumor pain, and transient hypertension were also observed. One dose limiting toxicity of nausea, vomiting, dehydration and neutropenia occurred. Drug-related neurologic toxicity or myelosuppression was not significant. Importantly, the combination of plinabulin and docetaxel did not increase their respective toxicities [25,184]. These phase I trials also demonstrated its antitumor activity. Plinabulin monotherapy achieved stable disease with a rate of 30% after 2 cycles [184]. Its combination with docetaxel led to a partial response in two patients and decreased tumors in four patients among total eight evaluable patients with non-small cell lung cancer [25]. These results support its further clinical development of either monotherapy or combination.

## 5. Conclusions

With the successful clinical uses of angiogenesis inhibitors and marine-derived anticancer drugs for cancer therapy, the development of marine-derived antiangiogenic agents is attracting more and more attention. To date, dozens of marine natural products and their synthetic analogues have been shown to inhibit angiogenesis or to disrupt established blood vessels, and several of them are undergoing anticancer clinical trials with encouraging results. Moreover, with the increasing exploration of marine sources, new marine angiogenesis inhibitors will continue to be found and developed, which will possibly offer more choices to the clinic for cancer therapy in future. In particular, marine-derived antiangiogenic protein kinase modulators will be hoped to achieve greater development, based on the critical roles of either TKs or S/TKs in tumor angiogenesis and the tremendous marine resources yet to be developed.
